# Machine learning for normal tissue complication probability prediction: Predictive power with versatility and easy implementation

**DOI:** 10.1016/j.ctro.2023.100595

**Published:** 2023-02-10

**Authors:** Pratik Samant, Dirk de Ruysscher, Frank Hoebers, Richard Canters, Emma Hall, Chris Nutting, Tim Maughan, Frank Van den Heuvel

**Affiliations:** a Oxford University Hospitals NHS Foundation Trust, Radiotherapy Physics, Oxford, United Kingdom; b University of Oxford, Department of Oncology, Oxford, United Kingdom; c Maastricht University Medical Centre, Department of Radiation Oncology (Maastro), Maastricht, The Netherlands; d Institute of Cancer Research, Division of Clinical Studies, Sutton, United Kingdom; e Institute of Cancer Research, Division of Radiotherapy and Imaging, Sutton, United Kingdom; f Zuidwest Radiotherapeutisch Instituut, Physics, Vlissingen (Flushing), The Netherlands

**Keywords:** Normal Tissue Complication Probability, Treatment Planning, Radiotherapy, Machine Learning, Xerostomia, Head and Neck Cancer, Clinical radiobiology, NTCP, Normal Tissue Complication Probability, RT, Radiotherapy, OAR, Organ(s) at Risk, GMD, Generalized Mean Dose, LKB, Lyman Kutcher Burman, ML, Machine Learning, GD, Gradient Descent, AB, AdaBooost (aka Adaptive Boosting), LR, Logistic Regression, DT, Decision Tree, GB, Gradient Boost, DA, Dual Annealing, DE, Differential Evolution, DVH, Dose Volume Histogram

## Abstract

**Background and purpose:**

A popular Normal tissue Complication (NTCP) model deployed to predict radiotherapy (RT) toxicity is the Lyman-Burman Kutcher (LKB) model of tissue complication. Despite the LKB model’s popularity, it can suffer from numerical instability and considers only the generalized mean dose (GMD) to an organ. Machine learning (ML) algorithms can potentially offer superior predictive power of the LKB model, and with fewer drawbacks. Here we examine the numerical characteristics and predictive power of the LKB model and compare these with those of ML.

**Materials and methods:**

Both an LKB model and ML models were used to predict G2 Xerostomia on patients following RT for head and neck cancer, using the dose volume histogram of parotid glands as the input feature. Model speed, convergence characteristics and predictive power was evaluated on an independent training set.

**Results:**

We found that only global optimization algorithms could guarantee a convergent and predictive LKB model. At the same time our results showed that ML models remained unconditionally convergent and predictive, while staying robust to gradient descent optimization. ML models outperform LKB in Brier score and accuracy but compare to LKB in ROC-AUC.

**Conclusion:**

We have demonstrated that ML models can quantify NTCP better than or as well as LKB models, even for a toxicity that the LKB model is particularly well suited to predict. ML models can offer this performance while offering fundamental advantages in model convergence, speed, and flexibility, and so could offer an alternative to the LKB model that could potentially be used in clinical RT planning decisions.

## Highlights

• NTCP modelling is not clinically used despite its importance in patient wellbeing.
• The LKB model is a popular NTCP model but is numerically unstable and inflexible.
• Even in a best case scenario for LKB, Machine Learning outperforms it in predicting NTCP.
• We show this on real clinical data and a completely independent validation set.
• Machine learning models therefore offer a superior alternative to LKB modelling.

## Introduction

Radiotherapy (RT) is a front-line cancer treatment in both palliative and curative settings. However, a lingering clinical problem is that complications often arise in healthy organs at risk (OARs) following RT. Therefore, the minimization of normal tissue complication probability (NTCP) is a key motif in RT treatment plan development and assessment. Indeed, NTCP management while ensuring prescription dose delivery could be said to be the entire objective of RT itself [1].

While the accurate modelling of NTCP is an important metric in treatment plan evaluation, NTCP modelling has not yet evolved to the point where it is routinely used in treatment plan evaluation. There are several reasons for this. Firstly, clinical realities (e.g. the addition of concurrent chemotherapy to RT) can often violate inbuilt assumptions of models (e.g. that NTCP is determined by dosimetric features alone). Secondly, models can be complex to implement and test in a generalizable way, either due to a lack of suitable scripting libraries for base functions or the need to manually craft and test model loss functions during fitting. Lastly, it is often difficult to be confident that a model that performs well on a validation set from one center will perform comparably to predict at another center due to batch effects in treatment practices. Therefore, there is a need for the development of NTCP models that are generalizable, can consider a wide variety of features, are simple to implement via scripting, and that are validated across multiple centers.

Perhaps the most widely deployed model for NTCP prediction is the Lyman Kutcher Burman (LKB) model [[2], [3], [4], [5], [6], [7], [8], [9]], in which a generalized mean dose (GMD) is combined with a probit function to create an equation predicting NTCP, namely

(1) $$\mathrm{NTCP}=\frac{1}{2}\left(1+\mathrm{erf}\left(\frac{GMD-D_{50}}{\sqrt{2}mD_{50}}\right)\right),and\;GMD={\left(\sum D_{i}^{\frac{1}{n}}v_{i}\right)}^{n}$$

Here, $D_{50}$ is the dose at which there is a 50 % chance of complication, m is a slope parameter (typically found via fitting the dose response curve), and GMD is typically computed directly from the differential dose volume histogram, consisting of Dose-Volume pairs $\left(D_{i},v_{i}\right)$. n is a dose-volume dependence parameter of a tissue and so incorporates tissue seriality into the model [1]. This version of the GMD (which is what we use in this study) is equivalent to the uniform equivalent dose (EUD) reported in some other literature [1].

Taken together, the three fitting parameters of this equation are n,m and $D_{50}$. In the case where GMD can be well approximated as the mean dose, n can be set to 1 and the number of fitting parameters reduces to two. One of the main advantages of the LKB model is its simplicity and ease of implementation. In addition, there now exist organ specific values of n,m and $D_{50}$ that can be used without the need for a priori fitting procedures if it is necessary to build a quick model for NTCP estimation [5]. The LKB model also has generalizability across various toxicities, as the same 3 parameters need to be fit for all toxicities in the same procedure. For these reasons, the LKB model has seen widespread use in the literature.

However, there remain some important drawbacks of the LKB model that have prevented its use in clinical treatment planning workflow. Firstly, the LKB model is based around the GMD, and consideration of other patient and/or treatment factors (e.g., age, gender, smoking, concurrent chemotherapy) requires specific changes to the model which are not inherently built into the base prediction function [10]. This can mean that the simplicity of the model declines in the case where a single GMD is not in and of itself the only major predictive factor of NTCP. Secondly, the LKB model can be numerically ambiguous during the fitting procedure, particularly in the instance where n is determined by fitting instead of assumption. This essentially means that the model is quite susceptible to the initial guesses of parameters chosen and will often not converge on a best fit depending on these guesses. Lastly, the fit parameters can themselves be ambiguous depending on initial guesses chosen, even in the case where the model does converge [[11], [12]]. This means that two separate researchers can attempt to fit a dataset and find two separate sets of values for n,m and $D_{50}$. The primary reason for this is that in the error function of the LKB model during the fitting procedure is prone to local minima, and so many optimization algorithms can fail to find global minima of error when methods like gradient descent (GD) are employed. It is therefore clear that alternative models must be explored and developed if NTCP prediction is to inform treatment planning.

ML models are good candidates for alternatives to LKB for several reasons. Firstly, ML models are now widely available in various easy to use in open-source and user-friendly environments with rapid and optimized runtimes [13]. Secondly, ML models can more easily consider clinical factors other than dose in model prediction as they are built to take a variety of different types of input features (binary, categorical, numerical, etc.). Lastly, ML models can be constructed such that the error function is always convex (i.e. such that the model is guaranteed to converge).

Here we examine the LKB model's performance, generalizability, and convergence. We compare this performance to some common ML algorithms such as AdaBoost (AB), Logistic Regression (LR), Decision Trees (DT), and Gradient Boosting (GB). We perform this procedure on two independent datasets (acting as training and test sets) of head and neck cancer patients treated at different centers. We correct for batch effects where possible for all continuous numerical features. We then compare the LKB model's ability to predict G2 Xerostomia on the validation set and explore its performance and robustness in doing so. Lastly, we compare this performance to common ML algorithms available in the scikit-learn library in Python.

## Methods

We fit the LKB and ML models on a training and test set at independent centers. Fitting procedures are described in Supplementary material. We then examined the performance of the LKB model in terms of model convergence characteristics, uniqueness of parameters, loss function behavior, and (in the case of convergence) performance to predict late grade 2 xerostomia toxicity (defined as G2 Xerostomia persisting after 6 months post RT).

### Data

For training the models, we used the data from the prospective “OutcomeH&N” registry of head and neck cancer patients at Maastro Clinic [[14], [15], [16], [17]]. For testing, we used data from patients in the multicenter PARSPORT trial (ISRCTN: 48243537)[[18], [19], [20], [21]]. Toxicity was assessed based on grade at 6-month follow-up after treatment. After applying corrections (Supplementary materials) for missing data, the final number of training set patients was 194, and the final number of test patients was 76. A statistical overview of the data is provided in the Supplementary material.

### ML and LKB model fitting

Both ML and LKB models were fitted to the training set, using a log-loss function in all models with the exception of DT (where Gini impurity was used instead). LKB optimization was performed using the scipy toolbox, feeding in the loss function to a minimization algorithm. ML optimization was done using the scikit-learn toolbox and the accompanying methods. For ML models, various model specific hyperparamaters (Supplementary Table 1) were tuned prior to model fitting using the roc-auc on validation folds. Full details of fitting procedure can be found in the Supplementary materials. Model assessment and comparison was assessed using the Brier score, as this is both a strictly proper scoring rule and an independent metric not used during the fitting process which is robust to prediction probabilities of 0 and 1.

**Table 1:** Classification Accuracy, ROC-AUC, and Brier score of all fit models on the training and testing datasets, time taken for hyperparameter tuning and fitting on the training set. ML models outperform LKB to classify G2 Xerostomia in the test set (but perform comparable in ROC-AUC). LKB: Lyman-Burman Kutcher; AB: AdaBoost; LR: Logistic Regression; DT: Decision Tree; GB: Gradient Boost; GD Gradient Descent; DA: Dual Annealing; DE: Differential Evolution.

Model	Accuracy		ROC-AUC		Brier Score		Tuning Time (s)	Fitting Time (s)
	Training	Test	Training	Test	Training	Test		
LKB	0.86	0.68	0.78	0.74	0.108	0.24	N/A	GD:0.667; DA:52.5; DE:3.37
AB	0.88	0.84	0.79	0.73	0.124	0.158	162	0.0699
LR	0.87	0.83	0.79	0.76	0.129	0.171	0.317	0.0024
DT	0.87	0.84	0.81	0.77	0.134	0.158	63.7	0.0031
GB	0.88	0.83	0.95	0.71	0.124	0.171	99.2	0.0434

### Batch effect correction

As our models are of most interest when deployed across centers, it is important to address the potential for center specific factors in the data during the fitting process. To address these, we have deployed the ComBat Algorithm [[22], [23], [24], [25], [26]] to correct for potential center effects. As shown in Supplementary Fig. 1, there are batch effects present between our training and test data, including with the important parameter of mean dose to the parotid gland. At the same time, to ensure model generalizability across centers, care must be taken such that the test set is as independent as possible to the training set (ideally from a difference center). Therefore, batch effect correction was necessary.

**Fig. 1:**
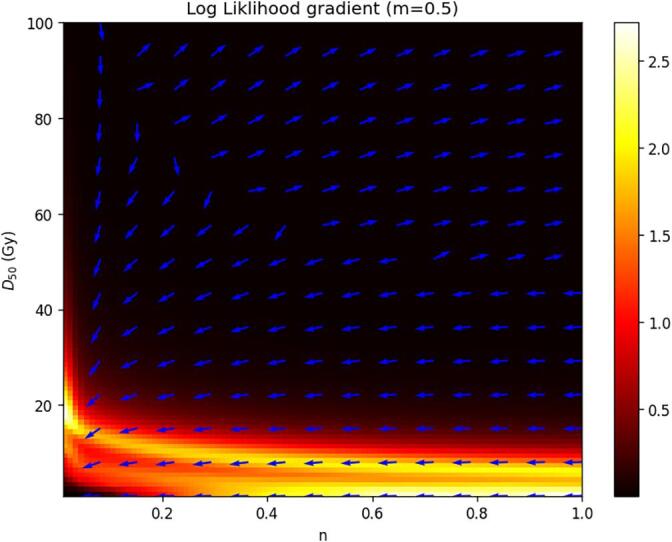
Gradient direction (blue arrow) and magnitude (colour map) as $D_{50}$ and n are varied. Depending on choice of initial guess, the gradients will lead in opposite directions. This behavior is the likely cause of GD failing in several instances to fit the LKB model. (For interpretation of the references to colour in this figure legend, the reader is referred to the web version of this article.)

Center specific effects can be treated similarly to batch effects in microarray expression because the ComBat algorithm’s transformation correcting for batch effect is mathematically similar regardless of the source of variation. Therefore, ComBat is well suited to correct for these effects as has been done in other studies [[22], [25], [26]].

## Results

### LKB convergence

The LKB model was found to be poorly suited for a GD based algorithm, and so would at times fail to converge depending on initial parameters. In trying 1 million initial values, 27.36 % of initial estimates failed to converge, and 28.63 % of initial estimates failed to converge to a predictive model (defined as a model with ROC-AUC ≥ 0.7). In the cases where a predictive model was achieved, the vast majority (99.34 %) of these cases the ROC-AUC was 0.74.

In both the cases where a global optimization algorithm (dual annealing or differential evolution) was used, convergence was achieved, however the runtime for convergence was several times that taken for the GD case. The optimal parameter set had some variation in the case of GD, but most predictive instances converged to values of n ∼ 1, m ∼ 0.55, and$D_{50}$ ∼ 47 Gy.The problem with deploying GD oriented algorithms is that the gradient of the cross-entropy does not lead to a well-established global minimum. This is illustrated in Fig. 1, where the gradient of the cross-entropy function is plotted as a function of n and $D_{50}$. Depending on the initial guess chosen by a GD algorithm, the direction of GD could point in two different directions. Similar behavior was observed keeping n and $D_{50}$ constant.

Global optimization was initially unsuccessful with the bounds specified with both dual annealing as well as differential evolution. This was traced to the set bounds on n, which allowed for a value as small as 0.001. Similar failure was observed allowing n (as in the case of m) to range from 0 to 1. However, if the algorithm bounds could be set to avoid small values of n (e.g. n≥0.01) then both global algorithms converged to values generally matching that of GD. This came at the cost of computation time as global optimization algorithms are typically more computationally expensive than GD algorithms.

Ultimately, to guarantee model convergence, the LKB model should not be fitted using conventional GD with some initial guess due to the phenomenon shown in Fig. 1. GD can lead the model to local minima in which the model can be non-predictive, or alternately can lead it to fail to converge onto optimal values. For this reason, the LKB model demands the use of global optimization algorithms, which can add computational expense. Lastly, care must be taken when using global optimization that n is not allowed to become very small, as small values of n can lead to computational infinities during GMD calculation. In principle, this also applies to m, which is a multiplicand of a denominator in equation 1, but we did not have this issue when allowing for m = 0 in our bounds.

### ML model fitting

ML model fitting was achieved in all instances as sci-kit learn algorithms choose either convex loss functions or algorithmic procedures such that convergence is always achieved according to some criteria (minimization of convex loss functions, maximizing information gain, etc.). Optimal parameters were found, and the Brier score loss was used to compare models. The Brier score was chosen over ROC-AUC as our main metric to compare models, as ROC-AUC is insensitive to differences in prediction confidence; i.e. the ROC curve is the probability that a given positive datapoint will be classified above a given negative datapoint, with no consideration for the difference in model confidence. Using this metric to assess model performance, ML models outperform the LKB model. However, it should be noted that all models (ML as well as LKB) had comparable ROC-AUC. Excluding hyperparameter tuning, ML models were substantially faster than LKB models to fit to the data, as they have well behaved loss functions and GD algorithms often benefit from speed. Lastly, these models were also able to take advantage of the sci-kit learn toolbox, which has highly optimized implementations of these algorithms.

### ML comparison to LKB

The predictive ability on training and testing data for the LKB model and all ML models is summarized below in Table 1. Our main metric of assessment is the Brier score on the test set, and by this metric the ML models outperform the LKB model. This means that ML models, themselves more versatile than LKB in terms of input features, could be a superior alternative to the LKB model in NTCP quantification. All ML models perform well in predicting patient toxicity and the none of the models fail to predict toxicity. ML models also outperformed the LKB model in raw accuracy scores. However, when using ROC-AUC for scoring, ML models and LKB models perform comparably.

Fitting and hyperparameter tuning times for all models are shown in Table 1. The ML model hyperparameter tuning time (which essentially among to a model selection step) could take several minutes in the case of AB and more than a minute for DT and GB. LG on the other hand was faster than the LKB model to both tune and fit to the data parameters. All ML model hyperparameter tuning was performed with 20-fold cross validation, so if lower run-times are desired, the number of folds can be lowered. When comparing fitting times, all ML models are clearly superior to the LKB model, regardless of the optimization algorithm used when fitting LKB, by orders of magnitude. This improved performance while retaining predictive ability is a key advantage of ML models over LKB.

## Discussion

Here we have compared the performance of ML approaches to the LKB model about predicting Xerostomia. We have found that ML models generally outperform the LKB models in classification (except in ROC-AUC), with some additional advantages of superior accuracy, fitting speed, ease of development, and reliably convex loss functions.

### Model convergence

Gradient Descent, while being the fastest method of fitting the LKB model, cannot guarantee convergence depending on the initial points used. However, when the LKB model with GD does converge (which depends on choosing the right initial guesses), the resulting model is quick to fit and has a predictive ROC-AUC score on the testing data. This can be overcome by deploying global optimization algorithms, which do not rely on the direction of gradient descent, but these algorithms are more computationally expensive and so the fitting procedure takes a longer time in these cases. Additionally, when performing global optimization, care must be taken to avoid situations where the algorithm samples the function for very small values of n and (presumably) m, as these can lead to infinities during function evaluation. In effect, this means that to have confidence of a good fit that is not susceptible to local minima in the error function, the LKB model demands that it be fitting using a global optimization algorithm.

The inability of GD to guarantee convergence in an LKB model is also a potential limitation of it going forward, as the restriction to global optimization will ultimately mean that the fitting process takes an order of magnitude (or more) time to fit in a way that an optimal parameter set is always found. This is also reflected in the timings that we have measured of LKB fitting with global and GD fitting algorithms in Table 1. Even in the case of gradient descent converging, the analytic form of the loss function gradient is not known, and so must be numerically evaluated during the procedure; this process adds additional computational expense.

In contrast, all tested ML algorithms always converged, and indeed are partially designed to have convex loss functions or algorithmic optimization of some criteria (e.g. decision trees can choose branches to maximize information gain). This advantage allows for ML fitting to be performed using without the concern that the algorithm will either fail to converge or converge on sub-optimal parameters due to local minima in the loss function. With the advantage of being able to converge on a model reliably, ML algorithms can quickly find predictive model parameters as compared to the LKB model and are therefore quite suitable for fitting on large datasets.

### Model performance

As measured by the Brier score and classification accuracy, ML models outperform the LKB model in predicting G2 Xerostomia.

ML performance was good even though our training and test set is looking at G2 Xerostomia, which is a toxicity that is particularly well predicted by the mean dose to the parotid glands. The LKB model is a GMD based model, and therefore, the situation we have tested the LKB model under is one in which it should already be poised to do quite well. In other situations where there are additional predictive factors that can determine patient toxicity, the LKB model will not be able to take advantage of additional information beyond the dose to a particular contoured structure.

It should be noted that while ML models did outperform LKB in Brier Score and accuracy, they did not outperform LKB in ROC-AUC (full ROC in Supplementary Fig. 2). This can be explained by the fact that in our specific dataset, we have selected a toxicity (xerostomia) that is particularly well correlated with the mean dose to the parotid gland [[27], [28], [29], [30], [31], [32]]. For this reason, the GMD to a good approximation reduces to the mean dose, and so GMD itself predicts Xerostomia well. ROC-AUC also suffers as a metric in some ways, specifically that it is insensitive to differences in model confidence. ROC curve values are simply the probability that a positive value will be ranked higher than a negative value, with no consideration for differences in model confidence. This is yet another reason that Brier score (a proper scoring rule) was used for model assessement.

### Model speed

Typical runtimes for fitting of all ML algorithms were orders of magnitude faster than GD, the fastest fitting procedure for LKB. Model hyperparameter tuning, in contrast, took many minutes. However, this is essentially a model selection (and not a model fitting) step, so we did not consider it to be as relevant to algorithm speed as the time taken to fit the data. Nevertheless it should be noted that with the exception of LG, all ML algorithms did take < 1 min to tune hyperparameters with 20-fold cross validation. If faster performance during tuning is desired, then the number of cross validation folds can be reduced. It is to be noted that fitting time is not necessarily a major drawback if a good model is built, as model evaluation for prediction on new data is rapid after fitting. It should also be noted that timings of LKB fitting were comparable to ML when GD was used (though this did not guarantee convergence), but using global optimizers increased to 3 s or even 52 s fit time.

ML development was substantially faster, simpler, and required less troubleshooting as compared to LKB implementation thanks to well established, well optimized, and open source Python libraries specifically tailored for rapid deployment of ML models. This means that researchers should generally be more rapidly able to write and deploy code for ML model fitting as compared to the LKB model, which will need to be developed without the help of these tools. It is possible that these well optimized libraries are partially responsible for the superior ML fitting performance (in addition to the fact that the LKB loss gradient is not analytically known), and the ability to use them is a powerful advantage of ML models over LKB. The LKB model on the other hand had to be developed manually due to the lack of optimized libraries. SciPy did offer pre-written libraries for optimizers, and sci-kit learn provided an optimized evaluation of the loss function, but the NTCP calculation function had to be generated in-house and this process meant that the development time for the LKB model was longer than that of the ML models; the latter could be written in relatively few lines of code.

### Model versatility

One crucial advantage that the ML models enjoy over LKB is the wide versatility of input features that they can take into them. Whereas the LKB model is entirely dosimetry, ML models can very easily also use categorical data and any additional numerical features (e.g. age, gender, concurrent chemotherapy, BMI, WHO score, etc.) that could be predictive of patient toxicity. In the case of predicting G2 Xerostomia, this is not a major problem; Xerostomia is well predicted by the mean dose to the parotid gland, and models have been known to perform well using the mean parotid dose as an input [[33], [34], [35], [36]], although also other OAR’s have been associated with xerostomia, including the submandibular glands [[36], [37]] as well as the oral cavity [[36], [38]]. This means that the ML models can be similarly deployed to a wide variety of toxicities with only minimal changes required to both implementation code and the model itself.

ML models also enjoy important advantages over LKB in batch effect correction between centers. The main reason for this is that the LKB model takes in an entire differential DVH as an input feature (and so ComBat is difficult to apply), whereas the ML models take in numerical features separately (e.g. mean dose, max dose, etc.). Individual numerical features can easily be fed into the ComBat algorithm, whereas individual DVH’s cannot be. Therefore, correcting for batch effects is much simpler to do in the ML case than that of LKB input.

### Comparison to previous studies using ML

There have been several excellent studies already looking into NTCP quantification using ML models. Christianen et al. [[39], [40]] had success in deploying multi-variable logistic regression to predict swallowing dysfunction following chemoradiation. Similarly, Wopken et al. [[41], [42]] examined a least absolute shrinkage and selection operator (LASSO) model’s predictive power on NTCP for tube feeding dependence and demonstrated a successful result. However, these studies did not examine or contrast different algorithms, and the LKB model was not used as a comparator. Dean et al. [43] examined machine learning (ML) for NTCP modelling of severe acute oral mucositis in 351 patients incorporating spatial dose metrics following chemoradiotherapy. The best performing model (random forest classification) achieved an area under the receiver operator characteristic curve (ROC-AUC) as high as 0.71. In a separate study, Dean et al. also applied models to predict severe acute dysphagia [44] and had comparable success, with a penalized logistic regression model scoring an ROC-AUC of 0.82 on external validation. However, neither of these studies investigated late toxicity, nor was performance contrasted with the LKB model. Gabrys et al. [45] explored NTCP modelling of early, late, and long term xerostomia with ML and compared to parotid mean dose based models such as LKB. However, the validation was not performed across multiple centers, so batch effects count not be accounted for. Jiang et al. [46] were successful in developing models predicting acute Xerostomia, but did not examine late complications. Several studies have also looked into NTCP modelling of other cancer sites [[47], [48], [49], [50]], but as each cancer type has varying statistical characteristics and is not equally suitable for the same models, lessons learned from modelling of other sites do not necessarily transfer when predicting NTCP or TCP, nor did these studies generally contrast models with machine learning with an independent validation set from a separate clinical trial.

In contrast, our study has investigated the performance of the LKB model as compared to ML classifying across multiple centers and accounting for batch effects to predict longitudinal complication. We found that ML models can provide superior predictive power with several additional advantages, such as the ability to incorporate batch effect corrections, and simple integration of additional clinical factors into the model.

### Summary and future directions

We have shown that the gradient of the LKB model’s loss function is poorly suited for GD algorithms, as the direction of the gradient can point the model in multiple directions, in which convergence can fail or (in rare cases) the model can converge on poorly predictive parameters. We have also shown that global algorithms must be deployed to overcome this, at the cost of extra computation time. We have demonstrated that while the LKB model is predictive at predicting G2 Xerostomia across centers, it does not outperform ML models in ROC-AUC, and underperforms in Brier score and accuracy. ML models offer superior performance while also retaining advantages in fitting speed, accuracy, generalizability, and ease of development.

This study demonstrates that ML models can outperform LKB even in the case where the mean dose to an organ is highly predictive (i.e. where the LKB model is well suited to succeed). In cases where other factors can also influence the likelihood of a toxicity (e.g. concurrent chemotherapy, the presence of both serial and parallel organs near one another, the presence of HPV, etc.) the LKB model cannot easily consider these additional factors whereas ML models are quite robust to them. A natural potential area of future research then, is to investigate how the models perform in a situation where mean dose to a single organ is not in and of itself predictive, and numerous different factors can contribute to patient toxicity. Examples of such toxicities include 1) Dysphagia in head and neck cancer patients, where the dose to multiple organs can be a factor 2) Nausea in pancreatic cancer patients, where concurrent chemotherapy can also be a factor, and 3) any radiotherapy courses in which heart toxicity is a concern, as the heart itself is a combination of parallel (muscle) and serial (nerves) structures which is not well characterized by a single n dependent GMD.

These areas of further research provide an exciting opportunity to develop models that are truly generalizable and predictive of NTCP across cancer patients, and to allow for NTCP calculation to become reliable enough to be a factor in clinical treatment planning decisions.

## Declaration of Competing Interest

The authors declare that they have no known competing financial interests or personal relationships that could have appeared to influence the work reported in this paper.
