# Supplementary material for: Machine learning for normal tissue complication probability prediction: Predictive power with versatility and easy implementation
Source: Clin Transl Radiat Oncol. 2023 Feb 10;39:100595. doi: 10.1016/j.ctro.2023.100595 (PMC9984444; doi:10.1016/j.ctro.2023.100595)
Supplement: Supplementary data 1 [file mmc1.docx]

LKB Model fitting

The LKB model was fit on constructed DVHs from the Training set for the dose delivered to the parotid glands. The available training set features (V10,V20...V90) were used in combination with the mean dose in order to construct a differential DVH of the patient to be used as an input. Where patient DVH features were not available (even if mean dose was available), patients were excluded from the training set. This additional patient filtering meant that the LKB training set consisted of 176 patients.

The loss function (aka error function) used was the binary cross-entropy, $L\left( y,p \right)$

$$\begin{aligned} L\left( y,p \right)=\sum_{i} -y_{i}\log\left( p_{i} \right)+\left( 1-y_{i} \right)\log\left( 1-p_{i} \right)\#\left( 2 \right) \end{aligned}$$

Where $p_{i}$ is the model’s probability estimate of the $i^{th}$ patient having toxicity, $y_{i}$ is whether the patient actually did have said toxicity (1 for yes, 0 for no), and the sum is performed over all patients. Ultimately, all our model fitting consisted of attempting to find values of $,n, m$ and $D_{50}$ that would minimize equation $2$.We attempted fitting via a bounded Gradient Descent (GD) algorithm (L-BFGS-B), an evolutionary algorithm (differential evolution) and a stochastic algorithm (simulated annealing). The bounds used were $n$ and $m$ between 0 and 1, and $D_{50}$ between 1 and 100Gy.

L-BFGS-B has some initial guess parameters that can affect the final values, so we examined the impact of initial guesses on algorithm performance in this case. We examined model convergence in the case of 1 million initial guesses, consisting of all possible combinations of the following parameters: 100 evenly spaced values for each $n$ and $m$ ranging from 0.01 to 1, and the same for $D_{50}$ ranging from 1 to 100Gy. The purpose of trying multiple initial guesses was to assess model stability as a function of initial conditions, as well as performance and generalizability in the case of convergence.

When model convergence was achieved, the model was then evaluated on the test set DVHs to evaluate the ROC-AUC. Optimization (using L-BFGS-B) was performed using SciPy’s ^1^ optimize toolbox with bounds of 0.001 to 1.0 on $n$, 0 to 1.0 on $m$, and 1.0Gy to 150.0Gy on $D_{50}$. Other metrics such as run-time for fit and performance on the test set were also collected.

ML Model fitting

Input features used in the ML models were DVH metrics of the parotid glands (Mean dose, Minimum Dose, Volume (cc), DMax, V10,V20...V90, etc.) as well as the patient gender and age. Additional demographic features of interest were also available in this dataset, however only gender and age were used as these were the only additional features simultaneously available in the test set.

From the training set, DVH's were extracted from patient RT-Struct and RT-Dose DICOM files imported into CERR ^2,3^. From CERR, features of interest matching those of the training set were extracted from the raw DVHs, along with patient age and gender.

We were able to correct batch effects with ComBat prior to ML model training^4^, however it is to be noted that this was not possible in the case of the LKB model. This is because batch effect correction via ComBat is done on set of numerical features on a feature-by-feature basis, but one of the inputs to the LKB model is an entire differential DVH. Therefore, only our ML models used data corrected for batch.

Next, we performed data scaling, principal component analysis (PCA), and hyperparameter tuning on ML models. Data scaling was done by subtracting the mean from all values and dividing the result by the standard deviation. We did this to both the training and testing data using the mean and standard deviation of the training set. We then optimized model hyperparameters using 20-fold cross validation according to the hyperparameter set shown in Supplementary Table 1, in addition to also tuning the number of PCA components (the number of components was chosen such that 10, 20, 30, 60, 80, 90, or 99 percent of variance was explained). Hyperparameter tuning was done to choose the model which maximized the ROC-AUC on the validation folds.

| Model | Hyperparameter set |
| --- | --- |
| AdaBoost | - Base Estimator: balanced and unbalanced cases of LR and DT were tried: - Number of Estimators: 5,10,20,40,80,160,320,450 |
| Decision Trees | - Minimum samples per leaf (as % of data): 0.01,0.05,0.1,0.2,0.5 - Minimum number of samples required to split an internal node: 2,5,7,8,10 - Maximum depth: 1,3,5, 10, 15, 30,58 - Number of features to consider when looking for the best split: $\sqrt{n}$ and ${log}_{2}n$ where n is the number of features |
| Logistic Regression | - Class Weight: balanced or unbalanced - Solver: liblinear or Newton-CG (where penalty allows) - Penalty: L1, L2, or none |
| Gradient Boosting | - Number of Estimators 20,40,80,160,320,640 - Fraction of samples to be used for fitting the individual base learners: 0.2,0.5,0.8,1 |

Supplementary Table 1: Hyperparameter tuning values used for ML fitting. In addition to all parameters shown above, the number of PCA components was also tuned for all models, keeping 10, 20, 30, 60, 80, 90, or 99 percent of variance.

Following hyperparameter tuning, the best performing models for each algorithm were then fit on the test set and evaluated on their ability to assess G2 Xerostomia. The ROC-AUC, Brier Score, and accuracy were assessed to check model performance.

**Data Processing and Description**

For the few cases where 6-month follow-up data was missing, data at a later follow-up (e.g. 12 months) was used where possible. Otherwise, if no data was available at 6 months or later, the patient was discarded from the set. Patients where a joint DVH could not be constructed for both parotid glands were also excluded.

We summarize some data characteristics below in Supplementary Table 2. Here it can be seen that several dose characteristics vary between the datasets, as well as the fraction of patients receiving primary RT. The % of patients receiving primary RT was quite different between training and test sets, and this could be a potential explanation for batch effects. As both datasets collected many features, we cannot be certain as to the root of the batch effects, but can nevertheless conclude that they are present.

|  | Age | Gender (% Female) | Average Dose to Parotid Glands across all patients(Gy) | | | Primary RT % |
| --- | --- | --- | --- | --- | --- | --- |
|  |  |  | Range (Max-Min dose) | Median | Mean |  |
| Train | 62.64 | 0.29 | 51.18 | 16.65 | 17.22 | 0.97 |
| Test | 58.16 | 0.275 | 52.18 | 42.09 | 46.54 | 0.74 |
| Train Xero+ | 62.46 | 0.23 | 61.3 | 28.61 | 27.79 | 1 |
| Train (Xero-) | 62.6 | 0.29 | 49.6 | 15.05 | 15.58 | 0.94 |
| Test (Xero+) | 56.75 | 0.08 | 51.45 | 59.02 | 57.34 | 0.5 |
| Test (Xero-) | 58.99 | 0.31 | 49.5 | 40.23 | 44.52 | 0.78 |

Supplementary Table 2: Statistical characteristics in training and test dataset

A visual representation of this phenomenon is shown in Supplementary Figure 1, in which boxplots of the mean dose to patients in the data are shown. It can be seen from the raw data that that there are some noticeable batch effects between the training and test datasets, and that the mean dose is predictive of xerostomia incidence in both these datasets.

For this reason, it seems that accuracy (defined as % of samples correctly classified) could be improved if batch effects could be accounted for (but not ROC-AUC).Therefore, we also examine and present the datasets after the application of the ComBat algorithm.^4^ The ComBat algorithm corrects for batch effects by estimating their impact and then performing an altered version location/scale adjustment of a mean and variance by mapping data to an intermediate probability distribution. We deployed ComBat with the pyComBat package on all patient dose features prior to training any ML models, but were not able to do the same for the LKB model as the LKB model takes in dose volume pairs from the differential dose volume histogram as an input. This is because batch effect correction via ComBat is done on set of numerical features on a feature-by-feature basis, but one of the inputs to the LKB model is an entire differential DVH. The effect of correcting for batch effects is also shown in Supplementary Figure 1.


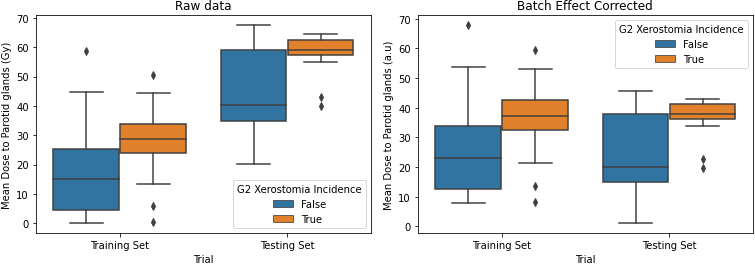


Supplementary Figure 1: G2 Xerostomia incidence in the data relative to the mean dose to the parotid glands before and after applying ComBat correction. Diamond shapes represent datapoints that are significantly different than the rest of the dataset. The box shape is the difference between the third and first quartiles.

While both the training and test set examined 6-month follow-up, the training set used CTC ^5^ and the test set used the LENT-SOMA ^6–8^ questionnaire. After examination and consultation with trial leads, it was decided that an appropriate agreement between these two datasets was that the CTC grade was one less than the LENT-SOMA Grade. i.e. CTC G2 Xerostomia = LENT-SOMA G3 Xerostomia, and we proceeded on this basis. When referring to G2 Xerostomia in this manuscript, we are referring to G2 or higher in the training (CTC) set, which corresponds to G3 (LENT-SOMA) in the test set.

Xerostomia incidence correlated well with the mean dose to the parotid glands (Supplementary Figure 1). This situation bodes quite well for the performance of the LKB model as this is primarily a model based on the GMD, and when the GMD (which reduces to the mean dose for n=1) is predictive, the model itself is also predictive. Therefore, this is an optimal scenario in which to deploy the LKB model.

ROC-AUC curves

For graphical visualization, the ROC curves on the testing set for all models is shown in Supplementary Figure 2, where predictive performance of the LKB model (in the case of convergence) is comparable to that of the ML models. Overall predictive performance was good as an ROC-AUC value of >0.7 is quite predictive. While all models do perform comparably, there is arguably a notable difference in performance between the best performing ML model (LR) and the worst performing ML model (GB). It should be noted that there were a very small minority (~1%) of LKB fits (with GD) which did successfully converge but had an ROC-AUC of ~0.5, so there was a chance of GD model convergence on values that would be non-predictive, but this issue could be avoided via global optimization algorithms. It should be noted (as shown in the main manuscript) that the Brier Score results show clear superiority of ML models over LKB for classification.

Supplementary Figure 2: ROC curves for all tested models. All ML models perform comparably to the LKB model on the test set.


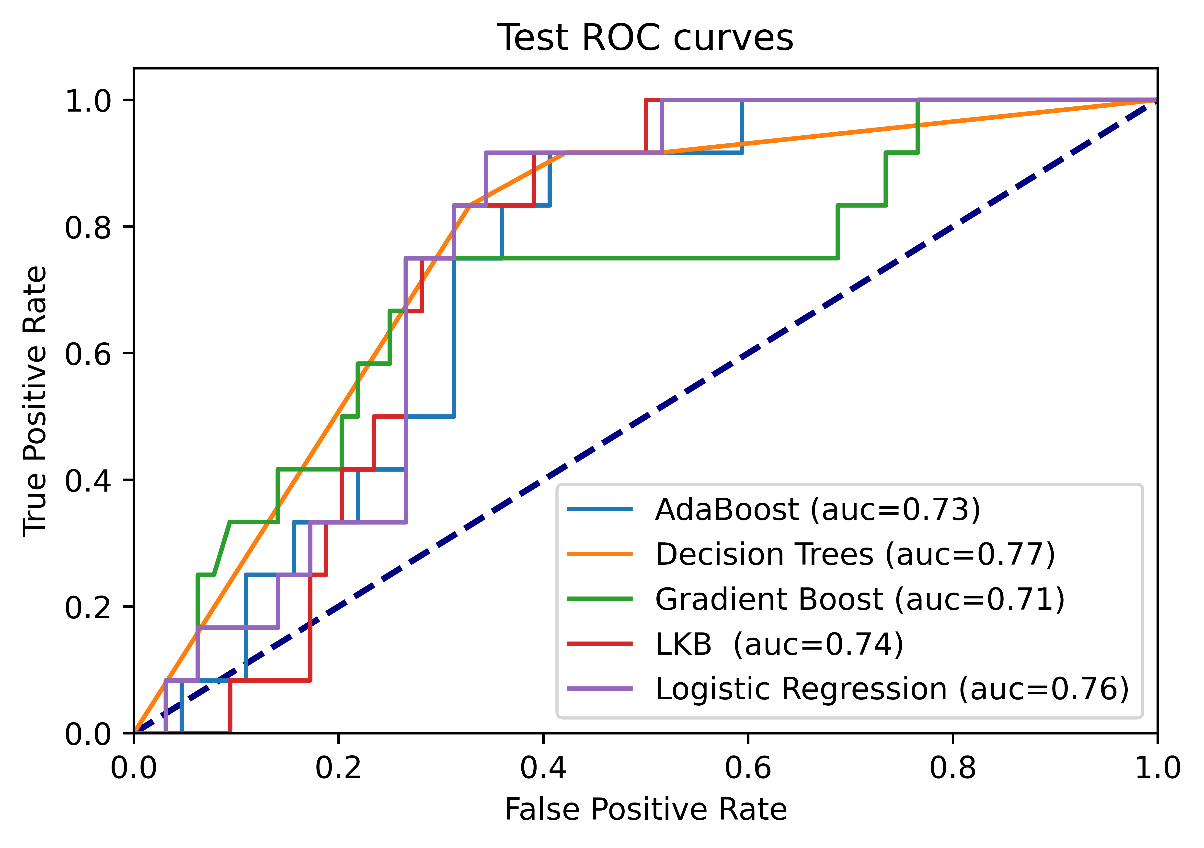


**Supplementary References**

1. Virtanen P, Gommers R, Oliphant TE, et al. SciPy 1.0: fundamental algorithms for scientific computing in Python. *Nat Methods*. 2020;17(3):261-272. doi:10.1038/s41592-019-0686-2

2. Deasy J. CERR: A Computational Environment for Radiotherapy Research. Published online 2014. https://ncihub.org/resources/594

3. Deasy JO, Blanco AI, Clark VH. CERR: A computational environment for radiotherapy research. *Medical Physics*. 2003;30(5):979-985. doi:10.1118/1.1568978

4. Johnson WE, Li C, Rabinovic A. Adjusting batch effects in microarray expression data using empirical Bayes methods. *Biostatistics*. 2007;8(1):118-127. doi:10.1093/biostatistics/kxj037

5. Blood Z, Tran A, Caleo L, et al. Implementation of patient-reported outcome measures and patient-reported experience measures in melanoma clinical quality registries: a systematic review. *BMJ Open*. 2021;11(2):e040751. doi:10.1136/bmjopen-2020-040751

6. Routledge JA, Burns MP, Swindell R, Khoo VS, West CML, Davidson SE. Evaluation of the LENT-SOMA scales for the prospective assessment of treatment morbidity in cervical carcinoma. *International Journal of Radiation Oncology*Biology*Physics*. 2003;56(2):502-510. doi:10.1016/S0360-3016(02)04578-9

7. Rubin P, Constine LS, Fajardo LF, Phillips TL, Wasserman TH. Overview of late effects normal tissues (LENT) scoring system. *Radiotherapy and Oncology*. 1995;35(1):9-10. doi:10.1016/0167-8140(95)97447-L

8. Pavy JJ, Denekamp J, Letschert J, et al. Late effects toxicity scoring: the SOMA scale. *Radiotherapy and Oncology*. 1995;35(1):11-15. doi:10.1016/0167-8140(95)97448-M
